# Dual Network Composites of Poly(vinyl alcohol)-Calcium Metaphosphate/Alginate with Osteogenic Ions for Bone Tissue Engineering in Oral and Maxillofacial Surgery

**DOI:** 10.3390/bioengineering8080107

**Published:** 2021-07-28

**Authors:** Lilis Iskandar, Lucy DiSilvio, Jonathan Acheson, Sanjukta Deb

**Affiliations:** 1 Centre for Oral, Clinical & Translational Sciences, Faculty of Dentistry, Oral & Craniofacial Sciences, King’s College London, Floor 17, Tower Wing, Guy’s Hospital, London SE1 9RT, UK; lilis.iskandar@kcl.ac.uk (L.I.); lucy.di_silvio@kcl.ac.uk (L.D.); 2 School of Engineering, Ulster University, Belfast BT37 0QB, UK; j.acheson@ulster.ac.uk

**Keywords:** bone tissue engineering, calcium phosphate, poly(vinyl alcohol), alginate, oral and maxillofacial surgery

## Abstract

Despite considerable advances in biomaterials-based bone tissue engineering technologies, autografts remain the gold standard for rehabilitating critical-sized bone defects in the oral and maxillofacial (OMF) region. A majority of advanced synthetic bone substitutes (SBS’s) have not transcended the pre-clinical stage due to inferior clinical performance and translational barriers, which include low scalability, high cost, regulatory restrictions, limited advanced facilities and human resources. The aim of this study is to develop clinically viable alternatives to address the challenges of bone tissue regeneration in the OMF region by developing ‘dual network composites’ (DNC’s) of calcium metaphosphate (CMP)—poly(vinyl alcohol) (PVA)/alginate with osteogenic ions: calcium, zinc and strontium. To fabricate DNC’s, single network composites of PVA/CMP with 10% (*w/v*) gelatine particles as porogen were developed using two freeze–thawing cycles and subsequently interpenetrated by guluronate-dominant sodium alginate and chelated with calcium, zinc or strontium ions. Physicochemical, compressive, water uptake, thermal, morphological and in vitro biological properties of DNC’s were characterised. The results demonstrated elastic 3D porous scaffolds resembling a ‘spongy bone’ with fluid absorbing capacity, easily sculptable to fit anatomically complex bone defects, biocompatible and osteoconductive in vitro, thus yielding potentially clinically viable for SBS alternatives in OMF surgery.

## 1. Introduction

Reconstruction of bone defects post tooth extraction, trauma, congenital deformities, removal of infective lesions, tumour or cysts, remains a challenge in OMF surgery [1]. Autografts continue to be the gold standard and thus remains the most frequently used bone substitute (~83% of total bone augmentation procedures) [2] despite the associated donor site morbidities and postoperative complications, such as prolonged pain, inflammation, neural dysfunction and infection, as well as the high cost due to prolonged surgery and hospitalisation period [3]. Synthetic bone substitutes (SBS) available to surgeons still mainly revolve around hydroxyapatite (HA), beta-tricalcium phosphate (β-TCP), calcium phosphate cement, calcium sulphate and Bioglass^®^ [4]. This is likely due to superior clinical outcomes of autografts and incomplete consideration in SBS design, which excludes translational aspects, such as simplicity in materials and fabrication methods, manufacturing scalability, ethical issues, and cost-efficiency.

Essentially, biomaterials for bone tissue engineering must be designed to maintain volume in the defect during the process of new bone tissue formation, enable transport of gases, nutrients, and regulatory factors, allow favourable cell-material interactions and biodegrade timely [4]. The OMF region raises special challenges for surgeons to manipulate the materials to fit irregular anatomical complexities due to complex neurovascular network, skin, subcutaneous tissues, salivary glands, and dental structures localised within a small area [5]. The proximity of OMF region to the oral cavities, nasal space and sinuses also increases risk of infections and decreases the outcome predictability of bone augmentation procedures [5]. Thus, the design of SBS for OMF surgery application should also consider sculptability, easy handling and antimicrobial properties.

Calcium phosphates (CaP’s) remain the mainstay of SBS due to their osteoconductive properties. However, they are brittle, lack ease of surgical manipulation and are unable to provide adequate fluid flow to reach the mid-section of a critical-sized defect to support cellular viability. Bioceramics-hydrogel composites have been trending for bone tissue engineering since they offer (i) malleable properties that greatly assist OMF surgeons to perform morphologically difficult procedures with often limited access to the defects; (ii) ability to absorb and retain biological fluids within the scaffold construct enabling perfusion of oxygen and nutrients necessary for cell survival, proliferation, differentiation, subsequent osteogenesis and vascularisation; and (iii) hydrogel nature, which enables concurrent delivery of cells, growth factors (GF’s), drugs, hormones, genes and other functional moieties [6].

The incorporation of biological products to enhance osteogenicity, however, may lead to adverse side effects, such as immunologic reaction, cytotoxicity and neoplastic induction [7]. Hence, incorporation of osteogenic ions, such as boron (B^3+^), calcium (Ca^2+^), cobalt (Co^2+^), copper (Cu^2+^), fluoride (F^−^), lithium (Li^+^), magnesium (Mg^2+^), niobium (Nb^5+^), phosphate (PO_4_^3−^), silicon (Si^4+^), silver (Ag^+^), strontium (Sr^2+^), vanadium (V^5+^), and zinc (Zn^2+^) may offer simpler, safer, and more cost-efficient alternatives for enhancing SBS properties [8]. Furthermore, some of these osteogenic ions, such as zinc, silver, and to some extent, strontium, have been reported to possess antimicrobial properties [9,10]. Compared to employing antibiotics, which raises concerns over antibiotic resistance [9], incorporating antimicrobial ions in SBS scaffolds provide a safer strategy for improving the success of bone regeneration, especially in areas prone to infection, such as the OMF region.

We earlier reported tough interpenetrating polymer network systems, referred to as dual networks (DN) [11] similar to double networks (DbN) [12]. The DN’s were formed using water-soluble polymers of poly(vinyl alcohol) (PVA) and alginate crosslinked via freeze–thawing (FT) of PVA and calcium salt solution chelation without incorporation of toxic solvents, initiators, activators and crosslinking agents. Combining the principle of forming DNs and our previous work on SBS based on single network composites (SNC) of poly(vinyl alcohol) (PVA) and calcium metaphosphate (CMP) [13], dual network composites (DNC) were designed to enhance clinical viability. The first network of the PVA-alginate DN was formed by FT of PVA solution and subsequently swollen in sodium alginate solution chelated with calcium chloride to form the second network. DN resulted in greater mechanical strength, toughness and ductility than its each polymer constituent due to the interpenetration of rigid alginate network into the PVA matrix, which increased the network density and stiffness, and the ability of the alginate network to break early and dissipate the energy, preventing rapid fracture growth [11]. Since divalent cations can be used to effect chelation of sodium alginate, osteogenic ions such as Ca^2+^, Zn^2+^ and Sr^2+^ were used to incorporate trace amounts of these ions in the PVA-CMP SNC. The chelation of alginate is a relatively easier method of doping ions compared to the more common methods, such as substituting Ca^2+^ in the calcium phosphate (CaP) crystal lattice [8], modifying the bioactive glass composition [14], or incorporating ionic salt particles into polymer composites [15].

In this study, SNC system was combined with DN system by using CMP particles as fillers within PVA matrix as the first network, interpenetrated with sodium alginate and chelated using osteogenic ions. Zn^2+^ and Sr^2+^ were selected due to their similar cationic valency with Ca^2+^ and their potential antimicrobial properties. Guluronate-dominant alginate (AlgG) was used instead of the mannuronate-dominant equivalent (AlgM) due to inability of Sr^2+^ and Ca^2+^ to chelate the MM blocks. Sr^2+^ ions only chelate with GG dimers whilst Ca^2+^ chelates GG and heterogenous GM/MG blocks of alginate [16]. These ionic affinity differences render AlgG with greater tensile, lower swelling and more stable thermal properties than AlgM [16].

## 2. Materials and Methods

### 2.1. Materials

β-CMP particles were produced using calcium bis-(dihydrogen phosphate) monohydrate (Ca(H_2_PO_4_)_2_·H_2_O) (CPMM) and PVA (Mw = 100.09 Da). DNCs were fabricated using PVA with Mw = 145 kDa, ≥98% hydrolysed, gelatine type A particles (porogen), sodium alginate from brown seaweed (*Lessonia flavicans*) (Kimica, Japan) with Mw = 53 kDa and M/G = 0.42, calcium nitrate tetrahydrate [Ca (NO_3_)_2_·4H_2_O], zinc sulphate heptahydrate (ZnSO_4_·7H_2_O) and strontium nitrate [Sr(NO_3_)_2_].

### 2.2. Dual Network Composite (DNC)

CMP was prepared by admixing CPMM and PVA in 4:1 weight ratio and heated in furnace as described earlier [13,17] and ground to particles with an average particle size diameter of ±14 µm. The SNC of PVA-CMP was fabricated as described by Nkhwa et al. [13]. In brief, a 10% (*w/v*) PVA solution and a slurry with CMP prepared at a ratio of PVA/CMP = 40/60 and 10% gelatine granules as porogen was subjected to two freeze–thawing cycles (2FT). Without washing out the porogen, SNC was immersed in 10% (*w/v*) sodium alginate solution for 48 h at room temperature then chelated in 0.1 M Ca/Zn/Sr salt solution for 24 h. The DNC was rinsed briefly with deionised water three times and subjected to one more FT cycle. Finally, the porogen and excess ions were washed out under agitation in deionised water at 37 °C for five days with water refreshed daily.

### 2.3. X-ray Diffraction (XRD) Spectroscopy

PANalytical X’pert PRO MPD high-resolution powder diffractometer with Co anode X-ray tube using CoKα1 radiation and incident beam Ge monochromator was used from 5° to 72° (2*θ* angle) with 40 kV tension and 30 mA current to scan diffraction spectra. X-ray diffraction patterns of all composites were collected to get crystal orientation data. For the display, the data were converted to CuKα1 spectra.

#### ^13^C-Nuclear Magnetic Resonance Spectroscopy (^13^C-NMR)

Samples were submerged into liquid nitrogen, ground into particles, and put into a cylindrical ceramic rotor. Solid-state cross-polarization magic angle spinning (CP/MAS) ^13^C-NMR was run using Bruker Avance TM 400WB with a wide-mouth superconducting magnet (89 mm) at 9.4 T (Larmor frequency 100.61 MHz). The ^13^C-NMR spectra of each sample were scanned with 4.40 µs width, 90 pulses, 3 s repetition, and 1 ms cross-polarization contact time for 4000 scans, referenced to adamantane and processed with MestReNova 12 software.

### 2.4. Attenuated Total Reflectance Fourier Transform Infrared Spectroscopy (ATR-FTIR)

ATR-FTIR absorbance spectra were obtained from dry scaffolds using Perkin Elmer Spectrum One spectrometer at 4000–650 cm^−1^ wavelength with 4 cm^−1^ resolution. All spectra were baseline corrected, smoothened (if required) and normalised using Spectrum software.

### 2.5. Raman Spectroscopy

Raman scattering spectra of dry samples were acquired using Renishaw Invia Raman spectrometer with 785 nm laser confocal microscope at 200–3200 cm^−1^ wavelength, 1200 L/mm grating, 10 s exposure time, and 1–5% laser power. Baseline correction, minor smoothening (if required) and normalisation of the spectra were conducted using Renishaw WiRE™ 5.2 software.

### 2.6. Compression Tests

#### 2.6.1. Static Compression Tests

Cylindrical specimens (8.5 mm diameter × 17 mm height) were prepared and tested in both dry and hydrated (pre-hydrated within deionised water for 24 h) states. A universal testing machine (Instron 5569A) with 5 kN load and Bluehill^®^2 software was used to collect and process the compressive stress, strain, and Young’s modulus at a crosshead speed of 1 mm/min. The ISO 291 (standard atmosphere for conditioning and testing) and ISO 604 (compression tests for plastics) were used as guidelines.

#### 2.6.2. Cyclic Compression Tests

All specimens were pre-hydrated in deionised water for 24 h. Cyclic compression tests to 40% strain with sinusoidal movement at 1 Hz frequency for 5 cycles with one-minute dwell time between cycles was conducted on a Bose ElectroForce 3300 testing machine. The compressive stress, strain, and Young’s modulus were recorded and calculated at each acquisition point using WinTest^®^ 7.0 software.

### 2.7. Water Uptake Study

Gravimetric measurements were conducted to determine the equilibrium water uptake on the specimens using at least 3 specimens for each group in deionised water at 37 °C. The equilibrium water content (EWC) was calculated:

$$EWC=\frac{Ws-Wd}{Ws}\times 100\%$$

*EWC*: equilibrium water content;

*Ws*: the weight of the hydrated sample at equilibrium;

*Wd*: the weight of the dry sample.

### 2.8. Differential Scanning Calorimetry (DSC)

15 mg of the dry sample (*n* = 3) in an aluminium pan (with hole) were scanned and compared to a blank aluminium pan in 20 mL/min nitrogen atmosphere using Perkin Elmer Jade DSC equipment (calibrated with Indium) using the thermal steps elaborated in Table 1. Glass transition (T_g_) (half C_p_ extrapolated) and melting (T_m_) temperatures were calculated from the curve in step#6 (Table 1) using Pyris™ 11.0 software.

### 2.9. Scanning Electron Microscopy (SEM)

Samples were mounted on aluminium stubs using conductive glue and coated with 5 nm gold prior to scanning under a vacuum using desktop SEM (JCM-6000plus, JEOL, Tokyo, Japan) with secondary electron detection mode (5–10 kV). Scanning electron micrographs were captured at regions of interest.

### 2.10. Micro-Computed Tomography (µCT)

Samples (*n* = 3) were scanned in hydrated state (pre-hydrated in deionised water for 24 h) using SkyScan 1275 (Bruker, MA, USA) X-ray microfocus CT rotating 360° at a pixel resolution of 6 µm, 45 kV peak voltage and 250 μA. The rotational images were converted to slices using Bruker’s NRECON 2.0 software. Slices were converted from greyscale to binary using a nominal threshold, which was applied to all samples in the study. Quantitative microstructural analysis was conducted using Bruker’s CTAn 1.18 software. All scans were reconstructed using the same reconstruction and threshold settings to ensure an accurate comparison.

### 2.11. In Vitro Biological Evaluation

Human osteoblast-like cells (HOS TE85) from European Collection of Authenticated Cell Cultures (ECACC) were used for cytocompatibility screening. Cells were cultured in Dulbecco’s Modified Eagle Medium (DMEM; 6046 SIGMA) containing 10% foetal calf serum (FCS) (Sigma F7524, non-USA origin), 5% HEPES, 1% minimal essential medium (MEM) (Sigma), 20 mM L-glutamines, and 1.5% ascorbate powder. Primary human bone marrow mesenchymal stem cells (hBMSC’s) (PromoCell C-12974) were used for investigation of osteoinductive potential of the scaffolds and were cultured in growth medium (PromoCell C-28009) from the same company. Cells were grown in a cell culture incubator with 5% CO_2_ level and 95% humidity at 37 °C. Test samples were sterilised by gamma irradiation (1.2 Mrad). Dynex Opsys MR™ colorimetric absorbance reader (570/620 nm) and Revelation software were used to quantify the optical data in MTT, protein and proliferation assays.

#### 2.11.1. MTT Assay for Cytotoxicity Evaluation

All in vitro biological evaluations were performed within the guidelines of ISO 10993-5 (in vitro cytotoxicity test) and ISO 10993-12 (sample preparation). Test samples (*n* = 4) were immersed in complete media (material/media = 0.025 g/mL) and agitated at 37 °C for 72 h to induce the release of any leachable substances (e.g., ions, unreacted monomer and oligomers). Cells were seeded in a 96-well plate (1 × 10^4^ cells/well) and incubated for 24 h. The media was removed and replaced by the test eluants (neat, dilutions of 50, 25 and 12.5%), DMEM with FCS as the negative control, and DMEM with FCS and 10% ethanol as the positive control. The cells were exposed to the eluants and controls for 24 and 72 h.

A sterile 5% (*w/v*) MTT (3-(4,5-dimethylthiazol-2-yl)-2,5-diphenyltetrazolium bromide) solution in phosphate buffer saline (PBS) was prepared and mixed with ascorbic-acid-free media at 1:10 solution to media. Following the designated exposure period, the eluant media were removed, and replaced with media containing MTT; cells were incubated for 2 h. The solution was then replaced by cell-culture-grade dimethyl sulfoxide (DMSO), and the plate agitated for 5 min to dissolve the MTT crystals, turning the solution purple. Colorimetric assay (570/620 nm) was used to quantify the relative cell viability.

#### 2.11.2. Protein Adsorption

Samples were pre-hydrated in PBS (pH = 7.4) for 24 h prior to immersion in 0.2 wt.% bovine serum albumin (BSA) in PBS under agitation at 37 °C for 30 min. The protein concentration was analysed with UV spectrometer (288 nm) using a standard curve. The adsorbed protein was calculated using following formula:

$$Adsorbed\;protein\;\left(\frac{mg}{g}\right)=\frac{Co-Ca}{w}V$$

*C_0_*: BSA concentration (mg/mL) before adsorption;

*C_a_*: BSA concentration (mg/mL) after adsorption;

*w*: weight of the hydrated samples (g);

*V*: volume of the BSA solution (ml).

#### 2.11.3. Alamarblue™ Cell Proliferation Assay

HBMSCs were micro-seeded on scaffold samples (10^5^ cells/scaffold) and incubated for 1, 3, 7, 14 and 21 days (*n* = 4). The alamarBlue™ was used to measure proliferation; the resazurin based assay is an oxidation-reduction (REDOX) indicator that undergoes colorimetric change in response to cellular metabolic reduction. The alamarBlue™ solution in phenol-red-free complete media was added at each time point, the plate was incubated for 4 h, and the alamarBlue™ solution was analysed using colorimetric absorbance assay (570/620 nm) for relative cell proliferation.

#### 2.11.4. Cell Lysate Preparation

Following the proliferation assay, HBMSC-seeded scaffolds were rinsed twice with PBS, immersed in distilled water (1 mL/well), then incubated (37 °C) and frozen (−80 °C) thrice to lyse the cells. These lysates were employed for total protein, alkaline phosphatase (ALP) and osteocalcin (OCN) measurements.

#### 2.11.5. RUNX2, BMP2, COL1A1, OPN and IBSP Gene Expression

The quantitative real-time polymerase chain reaction (qPCR) was used to determine the relative osteogenic gene expression of HBMSCs seeded on scaffold samples (±10^5^ cells/scaffold) at days 3, 7 and 14 (*n* = 3). The RNA was extracted using standard TRI reagent protocol. RNA concentration, quality, and purity were measured using nanodrop spectrophotometer (260/280 nm). The RNA was reverse transcribed to cDNA using iScript™ reverse transcription kit (BioRad, CA, USA) and Applied Biosystems Veriti thermal cycler following the kit guidelines. The qPCR used iTaq™ Universal SYBR^®^ Green (BioRad, CA, USA) and KiCqStart™ SYBR^®^ Green predesigned human gene primers (SIGMA KSPQ12012): glyceraldehyde-3-phosphate dehydrogenase (GAPDH) as housekeeping gene, runt-related transcription factor 2 (RUNX2), bone morphogenetic protein-2 (BMP2), collagen 1 alpha 1 (COL1A1), osteopontin (OPN) and bone sialoprotein (IBSP) as target genes (Table 2).

The gene expression was calculated using the relative standard curve method by testing a series of stock RNA dilution with known masses against each target gene. The quantified target gene was normalised to the housekeeping gene and calibrated to the target gene expression on CMP.

#### 2.11.6. Total Protein Production

Total protein was measured using Bio-Rad Bradford dye-binding protein assay kit. Colorimetric absorbance assay (590 nm) was used to acquire the optical data. The total protein concentration was calculated against the standard curve prepared using known concentrations of BSA mixed with Coomassie brilliant blue G-250 dye. The values were normalised to the relative proliferation of the same sample at the same time points.

#### 2.11.7. Alkaline Phosphatase (ALP) Production

A standard curve was prepared using 4-nitrophenol stock solution. Cell lysates were mixed with substrate reagent (p-nitrophenol phosphate, magnesium chloride hexahydrate, and triton X-100 in Glycine) at 1:1 ratio, incubated for 20 min, and the colour absorbance read at 405 nm. The ALP amount was normalised to the relative proliferation of the same cell source at the same time points.

#### 2.11.8. Osteocalcin (OCN) Production

Enzyme Linked-Immuno-Sorbent Assay (ELISA) kit (Invitrogen KAQ1381) was used to measure OCN production incorporating two positive controls, a blank, and negative control. The quantification was done using colour absorbance assay at 450 nm by interpolating from the prepared standard curve. The OCN production was adjusted to the relative proliferation of the same cell source at the same time points.

### 2.12. Statistical Analysis

Means and standard deviations (SD’s), normality tests, one-way ANOVA and post hoc Tukey’s analysis were done with GraphPad Prism 8 (confidence number * *p* < 0.05, ** *p* ≤ 0.01, *** *p* ≤ 0.001, and **** *p* ≤ 0.0001).

## 3. Results

### 3.1. Physicochemical Properties

**Figure 1:**
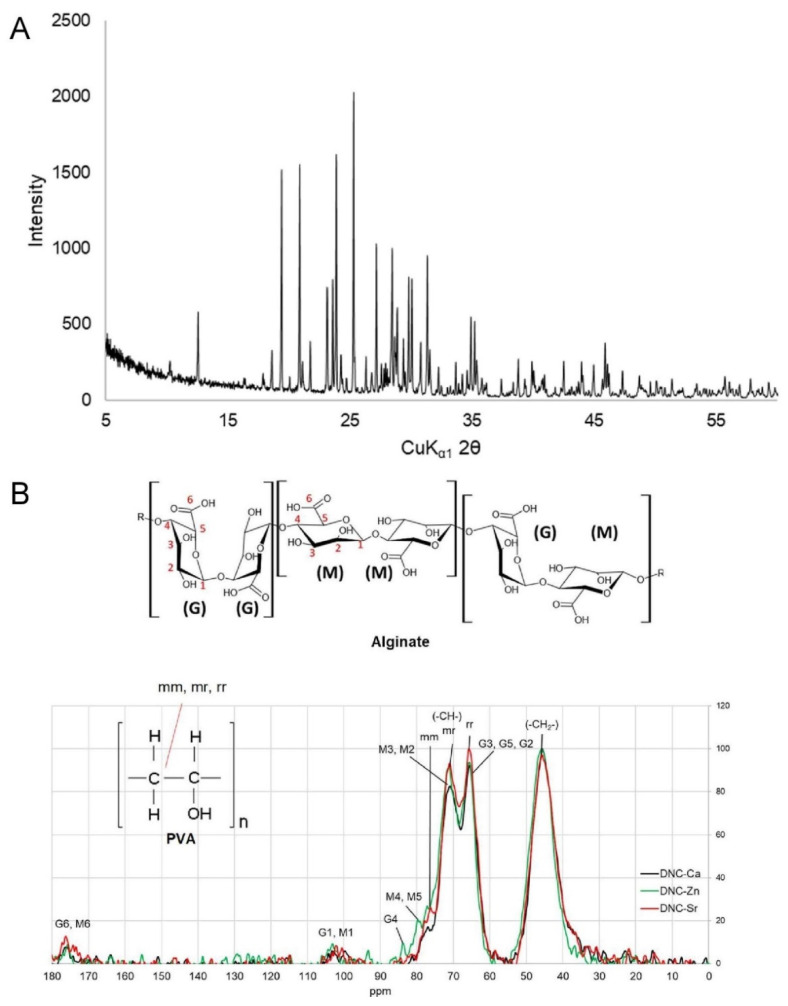

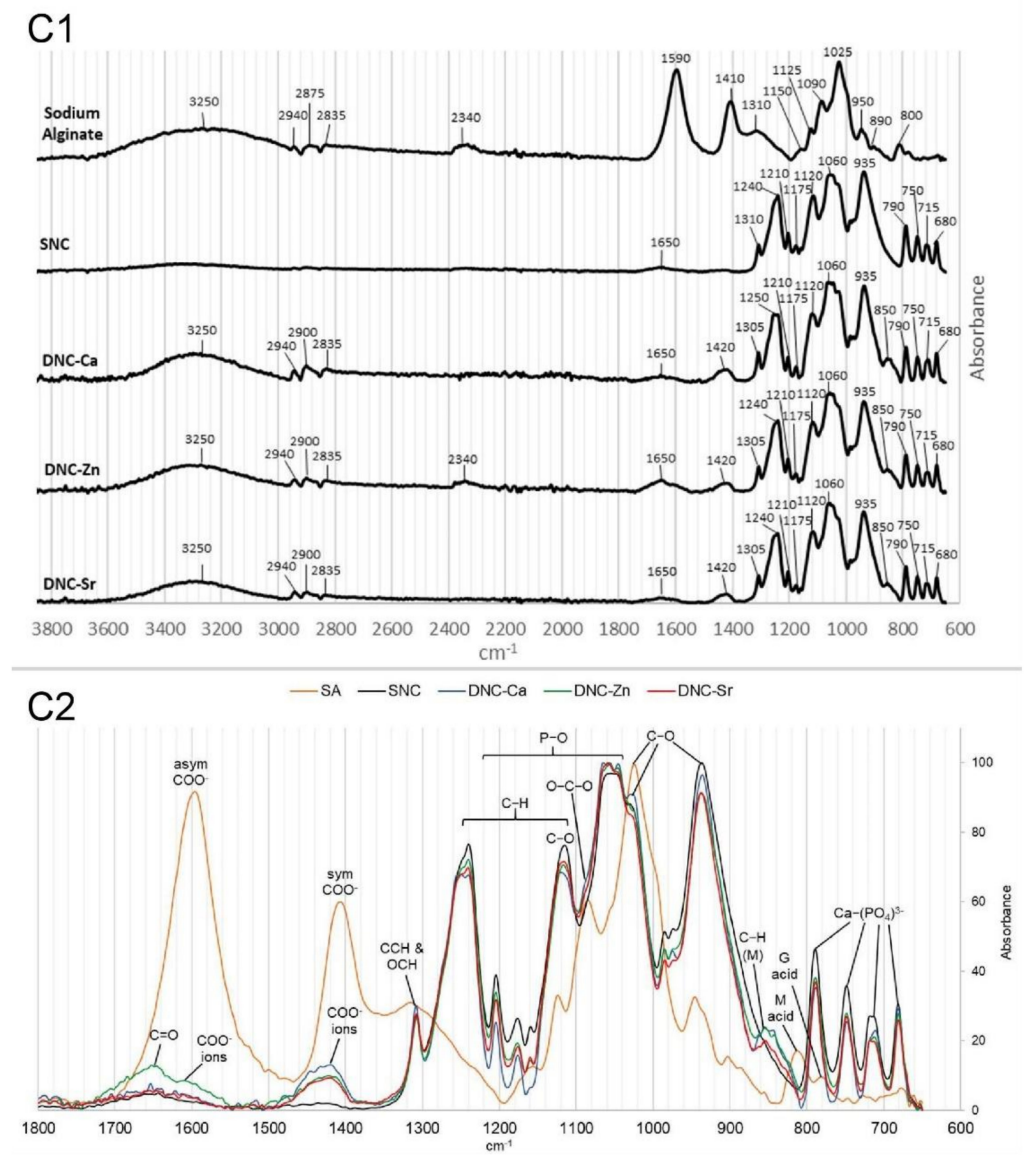

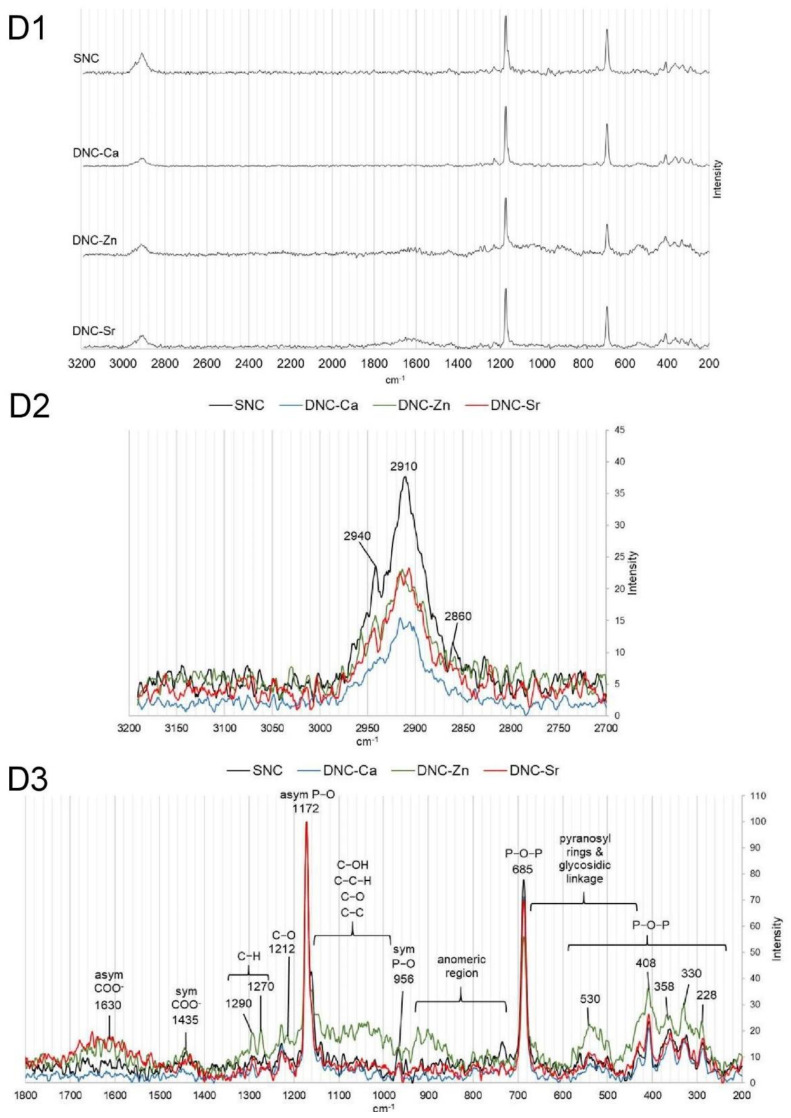
Dual network composites chelated with Ca^2+^ (DNC-Ca), Zn^2+^ (DNC-Zn) and Sr^2+^ (DNC-Sr): (**A**) X-ray diffraction (XRD) patterns, identical to those of β-CMP; (**B**) ^13^C-nuclear magnetic resonance (NMR) spectra; (**C1**) attenuated total reflectance Fourier transform infrared spectroscopy (ATR-FTIR) with full spectral range, (**C2**) stacked and expanded fingerprint region; (**D1**) Raman spectra with full spectral range, (**D2**) stacked, expanded peak and (**D3**) fingerprint region compared with the single network composite (SNC).

The XRD spectra of the DNC’s (Figure 1A) demonstrated identical XRD pattern to the composite filler, which is based on the International Centre for Diffraction Data (ICDD) database, comprises of β-CMP (Ca(PO_3_)_2_) (ICDD no. 01-079-0700) and catena-hexaphosphate or Trömelite (Ca_4_P_6_O_19_ or _4_CaO_3_P_2_O_5_) (ICDD no. 00-015-0177). This finding demonstrates that the process used to fabricate the composites did not alter the crystal phase of CMP.

The FTIR spectra of CMP, sodium alginate, DNC-Ca, DNC-Zn and DNC-Sr are shown in Figure 1C. The FTIR spectrum of CMP exhibited the characteristic peaks arising due to the phosphate groups with peaks due to the asymmetric stretching of the P-O bond of the PO_4_^3−^ at 1050–1200 cm^−1^ and oscillating vibrations between Ca^2+^ with PO_4_^3−^ at 650–800 cm^−1^ [19]. The broad peak centred at 3300 cm^−1^ arises from -OH stretching vibrations of PVA and alginates, whilst peaks around 2835–2940 cm^−1^ were attributed to the C-H stretching of PVA alkyl groups [20], the peak at 1650 cm^−1^ is due to C=O stretching of PVA acetate group [21], and the peak at 1310 cm^−1^ corresponds to -C-H and -OH vibration of PVA [21]. Overlapping peaks in the fingerprint region were attributed to (i) C-H wagging and twisting of PVA alkyl groups (1100–1250 cm^−1^), (ii) C-O vibrations of PVA hydrogen bonds (1115 cm^−1^) and (iii) P-O vibrations of the CMP phosphate groups (650–1200 cm^−1^) [13]. The presence of alginate also manifests in: (i) stretching of C-H anomer (2835–2940 cm^−1^); asymmetric and symmetric stretching of COO^−^ (1590–1605 cm^−1^ and 1410–1420 cm^−1^); bending of C-C-H and O-C-H (1305–1310 cm^−1^); C-O stretching (1120–1150, 1025–1030 and 935–950 cm^−1^); O-C-O ring shoulder (1060–1090 cm^−1^); C-H bending of mannuronate (800–890 cm^−1^) and guluronate (780 cm^−1^) [22].

The Raman spectra (Figure 1D) exhibited peaks correlated with CMP, which were previously invisible in IR spectra: (i) scissor bending of bridging oxygen in P-O-P (220–470 cm^−1^); (ii) deformational bending of P-O-P (500–580 cm^−1^); (ii) symmetric stretching of bridging oxygen in P-O-P (685 cm^−1^); (iii) symmetric stretching of non-bridging P-O (956 cm^−1^), and (iv) asymmetric stretching of non-bridging P-O (1172 cm^−1^) [23]. Whilst from PVA, peaks associated with C-H and O-H stretching vibrations were observed at 2850–2970 cm^−1^ [24]. The Raman peaks arising from the alginate are: (i) asymmetric stretching of COO^−^ (1500–1700 cm^−1^); (ii) symmetric stretching of COO^−^ (1400–1460 cm^−1^); (iii) C-H deformation (1250–1300 cm^−1^); (iv) C-O stretching (1200–1240 cm^−1^); (v) C-OH deformation, C-C-H bending, C-O and C-C stretching vibrations (1200–950 cm^−1^); (vi) anomeric region skeletal vibrations and ring breathing (950–750 cm^−1^); and (vii) pyranosyl ring deformation and C-O-C glycosidic linkage vibrations (410–690 cm^−1^) [25].

^13^C NMR spectra of DNC’s (Figure 1B) showed peaks arising from PVA and alginate. The peaks arising from PVA correlate to the methylene carbon (-CH_2_-) resonance at 46 ppm, and the syndiotactic (rr), heterotactic (mr) and isotactic (mm) triad of methine carbon (-CH-) at 66, 71 and 77 ppm [26]. The peaks from the alginate units correlate to the carboxyl group at 177 ppm (G6 and M6), anomeric group at 103 ppm (G1 and M1), and the pyranosyl ring groups at 60–87 ppm (G2, G3, G5, M2, M3, M4, M5 and G4), which overlap with PVA methine triads [27].

### 3.2. Compressive Properties

**Figure 2:**
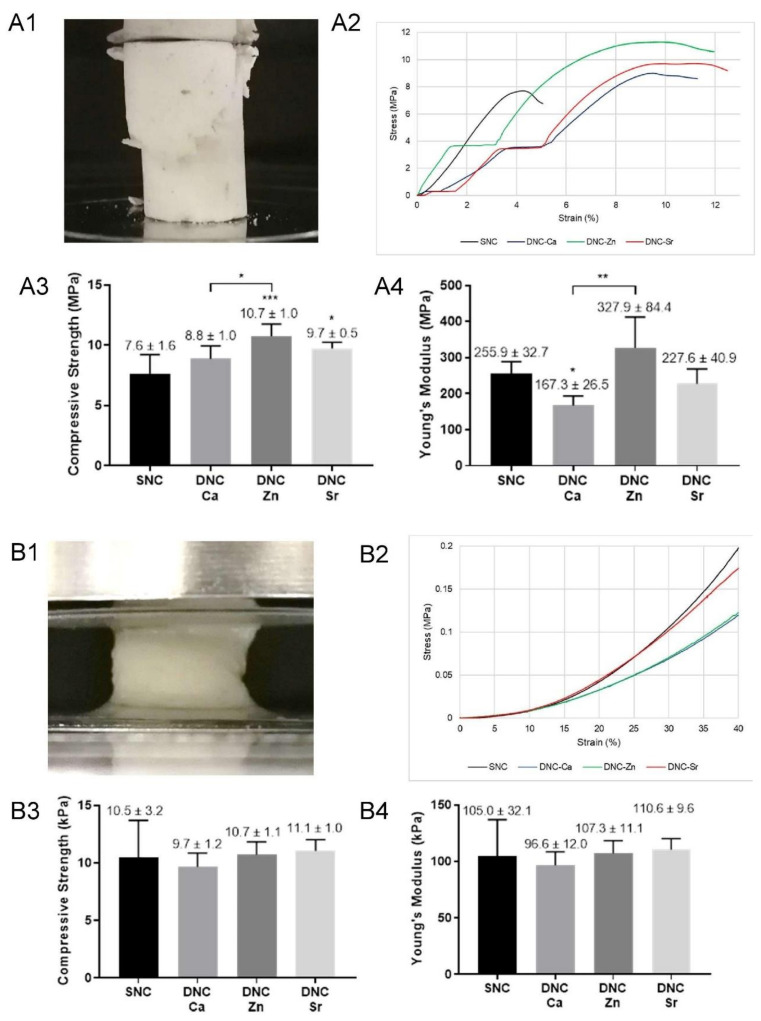

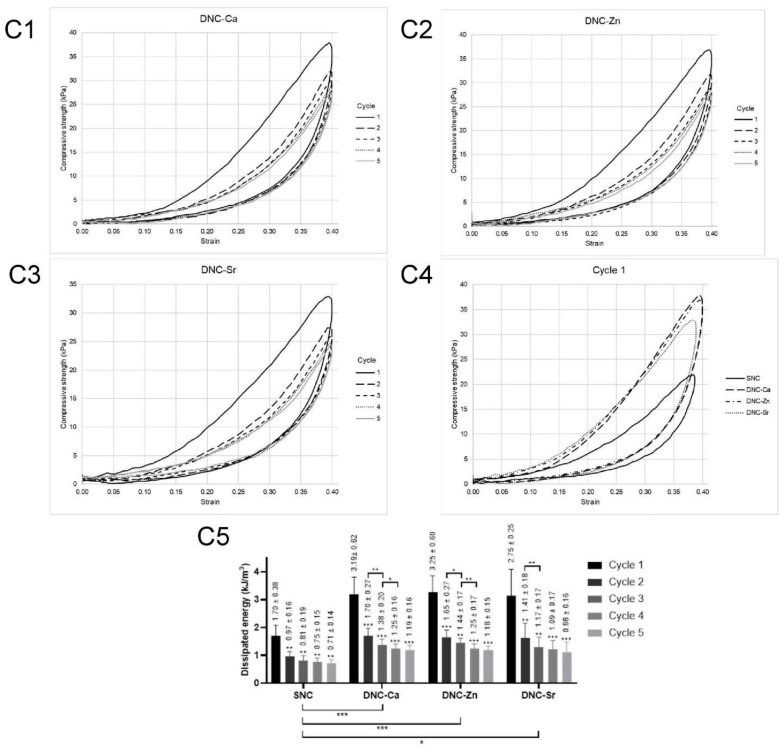
Dual network composites chelated with Ca^2+^ (DNC-Ca), Zn^2+^ (DNC-Zn) and Sr^2+^ (DNC-Sr) under static and dynamic compression compared with the single network composite (SNC): (**A1–4**) representative image, stress–strain curves, compressive strength and modulus in the dry state and (**B1–4**) hydrated state (at 10% strain); (**C1**) representative image, stress–strain curves up to 40% strain for five compressive cycles in the hydrated state of DNC-Ca, (**C2**) DNC-Zn, (**C3**) DNC-Sr, (**C4**) their comparison at the first cycle, and (**C5**) the dissipated energy at each cycle. Data are displayed as mean ± SD, bars represent means and error bars represent SD’s (*n* = 6). Statistical significance was verified with one-Way ANOVA and post hoc Tukey’s analysis (* *p* < 0.05, ** *p* ≤ 0.01 and *** *p* ≤ 0.001).

Dry DNC’s exhibited typical diagonal splitting compressive fracture (Figure 2(A1)), with the stress–strain curves showing multiple elastic and plateau regions, followed by plastic plateau region before fracture (Figure 2(A2)). Dry DNC-Zn demonstrated the highest compressive strength and modulus (Figure 2(A3,A4)).

In the hydrated state, DNC’s did not fracture until maximum compressibility (Figure 2(B1)), hence the compressive strength and modulus were recorded in the linear elastic region (Hookean) at 10% strain. In the stress–strain curves (Figure 2(B2)), a plastic plateau region did not exist; the curves of all DNC’s exhibit sharp increase, indicating structure densification, which created counter-resistance to the compressive force. Regardless of the ionic species, the DNCs in the hydrated state maintained the bulk integrity under compressive load with similar compressive strength and modulus (Figure 2(B3,B4)).

The cyclic compression tests (Figure 2(C1–C3)) exhibited hysteresis loops between the loading and unloading curves with DNC’s demonstrated greater hysteresis in comparison to SNC scaffolds (Figure 2(C4)). Although bulk fracture did not occur, the dissipated energy of the scaffolds significantly dropped following the first cycle (Figure 2(C5)).

### 3.3. Water Uptake

**Figure 3:**
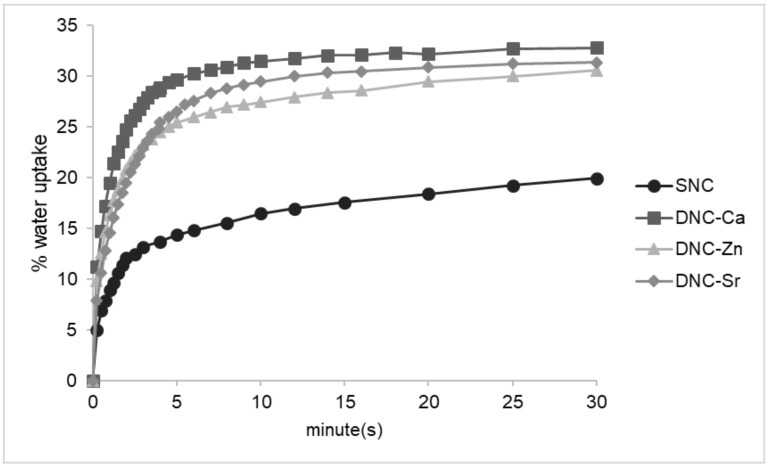
Water uptake of dual network composites chelated with Ca^2+^ (DNC-Ca), Zn^2+^ (DNC-Zn) and Sr^2+^ (DNC-Sr) compared to the single network composite (SNC) at initial period in deionised water at 37 °C.

The DNCs demonstrated controlled swelling with equilibrium water content (EWC) ranging between 37–40% with the presence of the second hydrogel network in DNCs resulted in higher swelling than the SNC equivalent (Table 3). The rate of water intrusion in the initial stages was also higher in the DNC scaffolds in comparison to SNC (Figure 3). A comparison of the DNCs with different ionic species showed that the water uptake rate of DNC-Zn was slower, followed by DNC-Sr and DNC-Ca.

### 3.4. Thermal Properties

The DNC’s exhibited a single glass transition temperature (T_g_) (Table 4) ranging between 132–135 °C. A comparison of DNC’s chelated with different ions did not show any statistically significant difference in T_g_. The melting temperature (T_m_) of the DNCs were similar to that of SNC (Table 4).

### 3.5. Morphological Properties

**Figure 4:**
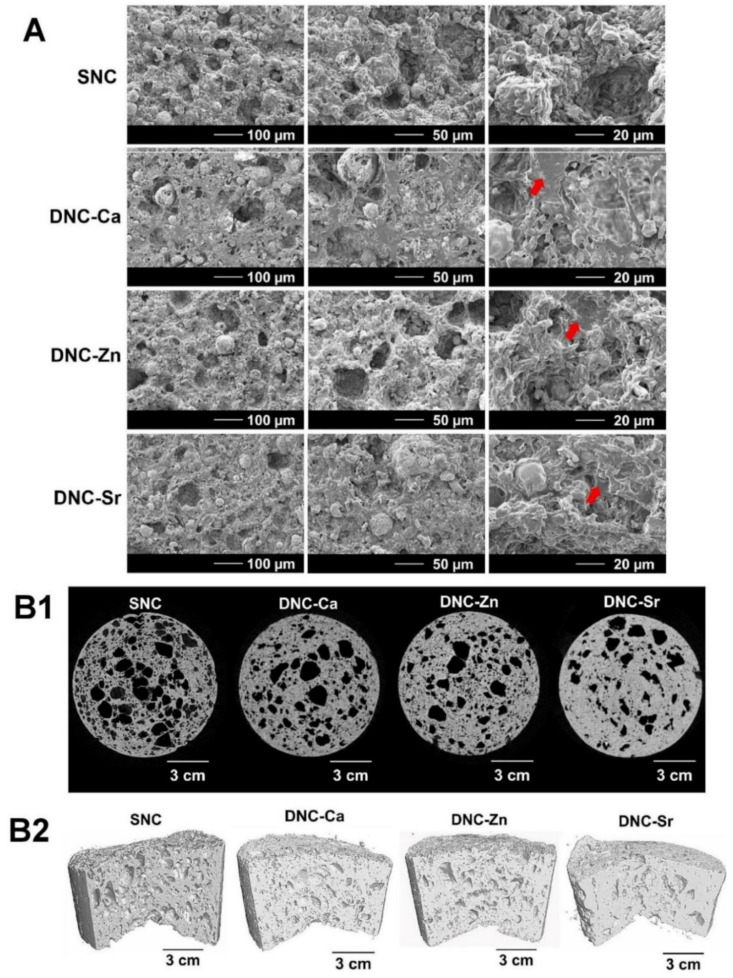
Morphological characterisation of the dual network composites chelated with Ca^2+^ (DNC-Ca), Zn^2+^ (DNC-Zn) and Sr^2+^ (DNC-Sr) compared with the single network composite (SNC): (**A**) scanning electron micrographs in the dry state. Smears of polymeric network are visible in the DNC’s (indicated by red arrows), likely to be the alginate network; (**B1**) micro-computed tomography (µCT) at cross-sectional and (**B2**) sagittal plane in the hydrated state.

The scanning electron micrographs show a homogenous distribution of CMP in the DNC matrix with macro- and microporosity (Figure 4A). Although a smear layer of polymeric network which was likely to be the alginate was visible at higher magnification, a seamless interface between the CMP filler particles and the polymer phase was evident.

In the 3D µCT images, DNC’s show evidence of porosity (Figure 4(B1,B2)) with a denser structure (Table 5) and lower total and open porosity (Table 5) compared to the SNC. Among the DNC’s, DNC-Ca exhibits a higher porosity than DNC-Sr or DNC-Zn. All DNC’s demonstrates near zero degree of anisotropy of the DNC’s (Table 5), suggesting that these materials are isotropic.

### 3.6. In Vitro Biological Properties

**Figure 5:**
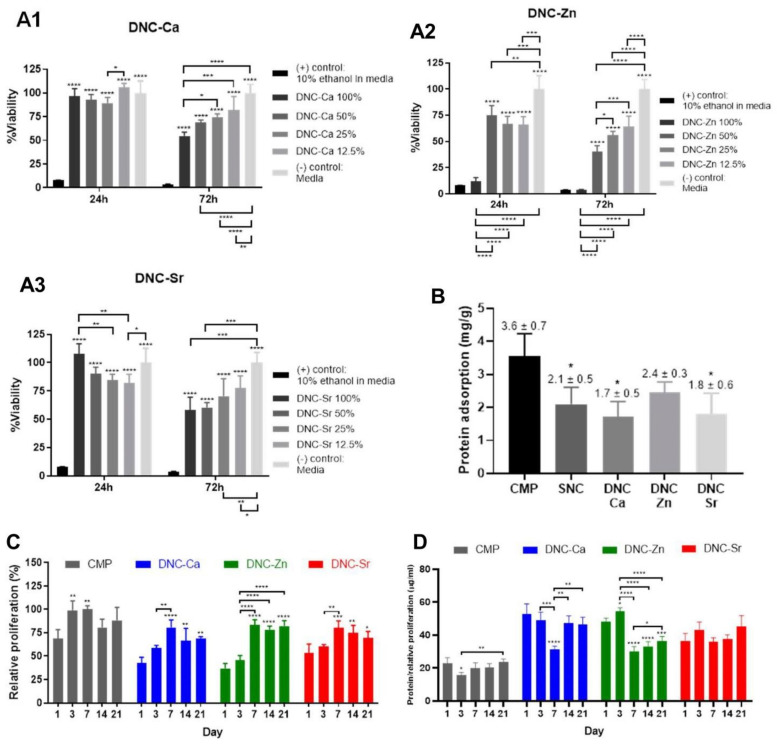

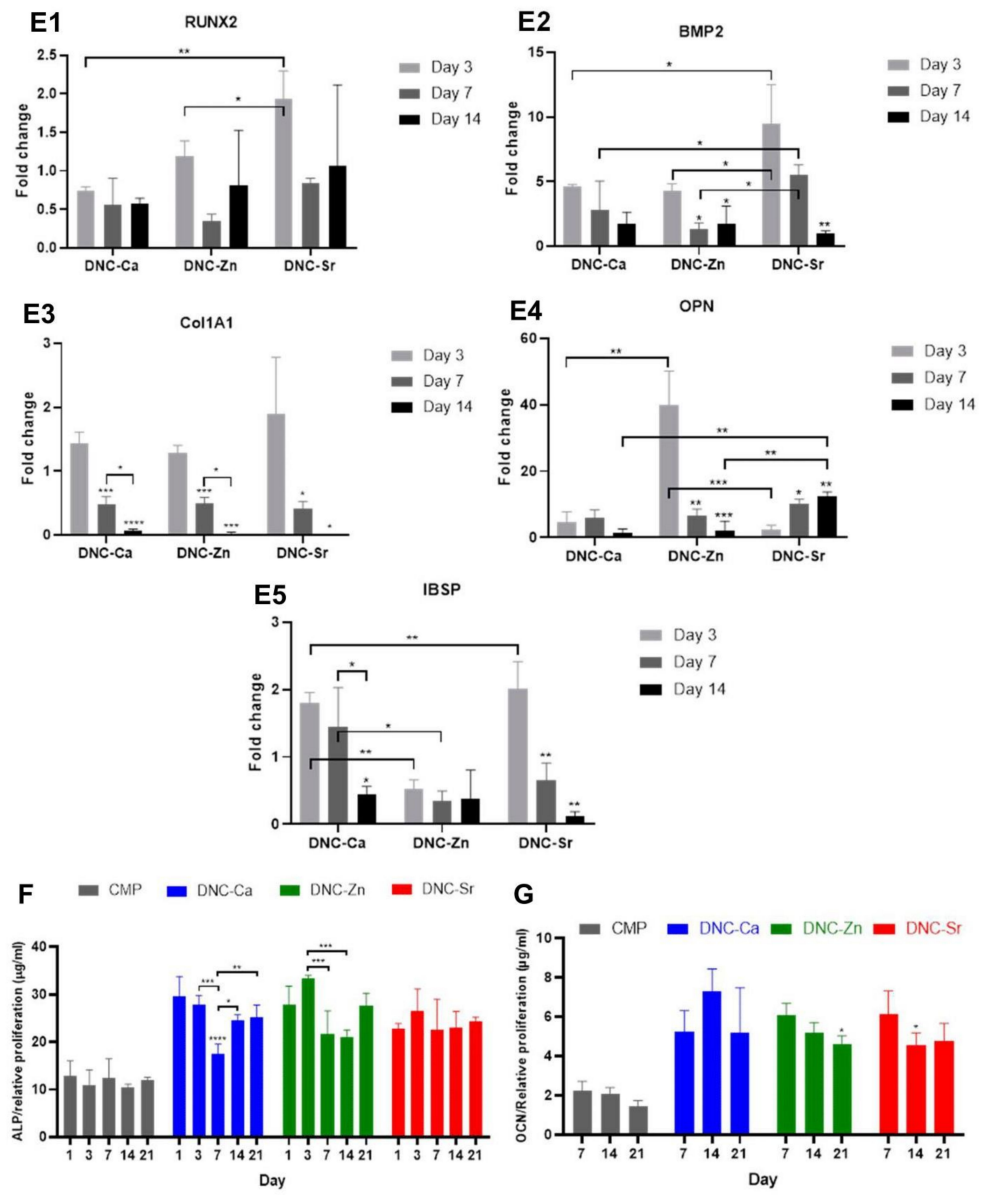
In vitro biological evaluation of dual network composites chelated with Ca^2+^ (DNC-Ca), Zn^2+^ (DNC-Zn) and Sr^2+^ (DNC-Sr): (**A1–A3**) human osteoblast-like cell (HOS TE85) viability following exposure to scaffold eluants; (**B**) bovine serum albumin (BSA) protein adsorption (mg/g); (**C**) human bone marrow mesenchymal stem cells (hBMSC’s) relative proliferation; (**D**) total protein production/relative proliferation; (**E1**) runt-related transcription factor 2 (RUNX2) (**E2**) bone morphogenetic protein-2 (BMP2), (**E3**) collagen 1 alpha 1 (COL1A1), (**E4**) osteopontin (OPN) and (**E5**) bone sialoprotein (IBSP) gene expression; (**F**) alkaline phosphatase (ALP) production/relative proliferation; (**G**) osteocalcin (OCN) production/relative proliferation. Bars represent means and error bars represent SD’s (*n* = 4). Statistical significance was verified with one-way ANOVA and post hoc Tukey’s analysis (* *p* < 0.05, ** *p* ≤ 0.01, *** *p* ≤ 0.001, and **** *p* ≤ 0.0001).

Figure 5(A1–A3) suggest non-cytotoxicity of DNC-Ca and DNC-Sr, but dose-dependent cytotoxicity of DNC-Zn eluants to HOS cells. The amount of adsorbed protein was higher on CMP compared to the composites (Figure 5B). Among DNC’s, DNC-Zn adsorbed higher amount of protein than DNC-Ca and Sr. HBMSC’s proliferation peaked at later time point on DNC’s (day 7) than on CMP (day 3) and became relatively stable afterwards (Figure 5C).

The presence of osteogenic ions was found to enhance the osteogenic gene expressions in HBMSC’s in varying ways. Ca^2+^, Zn^2+^ and Sr^2+^ ions increased the total synthesised protein per cell (Figure 5D), runt-related transcription factor 2 (RUNX2) (Figure 5(E1)), bone morphogenetic protein 2 (BMP2) (Figure 5(E2)), collagen 1 alpha 1 (COL1A1) (Figure 5(E3)) expressions at early period with gradual reduction over time. However, the osteopontin (OPN) expression was low on DNC-Ca at all time points and was highly expressed on DNC-Zn at early period and on DNC-Sr at later period (Figure 5(E4)). Whilst human integrin-binding sialoprotein (IBSP) was expressed at higher level on DNC-Ca and DNC-Sr at early period, but not on DNC-Zn (Figure 5(E5)). The presence of osteogenic ions was also found to increase alkaline phosphatase (ALP) and osteocalcin (OCN) protein productions by hBMSC’s on DNCs compared to CMP (Figure 5F,G). The ALP production on DNC-Ca and DNC-Zn was high until day 3, but relatively stable on DNC-Sr, whilst the OCN production was either stable across the time points or declined at day 14 or day 21.

## 4. Discussion

A new class of composite material based on dual network system [11] containing osteogenic ions of Ca^2+^, Zn^2+^ and Sr^2+^ was successfully developed. ^13^C-NMR, FTIR and Raman spectra of DNC-Ca, Zn and Sr demonstrated distinct broadening, shifting and peak intensity. These differences likely correlate with the ionisation potential, ionic charge, the mass and size of the ions, types of subatomic orbital participation, hydrogen bonds, and types of metal–carboxylate coordination [22], which account for the different affinity of the ionic species with alginate dimeric blocks [16]. Not only in the coordination with the alginate functional groups, DNC’s chelated with Ca^2+^, Zn^2+^ and Sr^2+^ also reflected distinction in the FTIR and Raman spectra for functional groups related to PVA and CMP. This finding suggests that the ion–alginate affinity differences also likely influence the strength of the intra- and intermolecular hydrogen bonds between each composite constituent since they are bound in close vicinity.

Uniaxial compression tests were conducted in dry and hydrated states. The multiple elastic and plastic regions in the stress–strain curves of DNC’s before fracture (Figure 2(A2)) can be associated with slippage of the specimens, but more likely due to polymer chain rearrangements which is not observed in SNC’s, indicating the effect of the second network formed by the alginate. The superior compressive strength and modulus of dry DNC-Zn (Figure 2(A3,A4)) were likely due to the non-selective binding ability of Zn^2+^ to all alginate dimers (GG, MM, GM/MG) [16], which renders the DNC-Zn network homogenous and denser, yet flexible to resist greater compressive load. In the hydrated state, PVA/alginate DN matrix is more flexible than a single network of alginate, as seen in the comparison with a similar study by Zamani et al., hydrated alginate–bioglass scaffolds showed an initial linear elastic region of the stress–strain curves followed by a plateau region, which indicated plastic deformation [28].

In cyclic compression tests, hysteresis loops between the loading and unloading curves indicated the ability of the scaffold to dissipate energy, which arise from the friction between polymeric chains and buckling of the scaffold internal microstructure [29]. DNC’s greater hysteresis than the SNC (Figure 2(C4)) suggested a more efficient energy dissipation [30], which is likely due to the synergy of PVA and alginate interpenetrating network. This synergistic effect was found to be similar to a study by Sun et al. using alginate and polyacrylamide where the energy dissipation was also higher in the hybrid network [30]. The first compressive load likely caused irreversible internal microstructural fractures due to the sacrificial bonds having been completely impaired in the first cycle and the healing period for the scaffold was insufficient. To heal from fatigue damage, hydrogen bonds were reported to require 1–4 h rest at room temperature [19]. Ca-alginate crosslink that may rupture can undergo partial healing when incubated at 60 °C for minimum one day [30], but are irreversible at room temperature [19]. However, after the first cycle, the DNCs demonstrated stable energy dissipation and structural integrity under multiple compressive loads, which deems them suitable for use in area subject to functional loads, such as the OMF region.

The low EWC of DNCs is favoured as extreme swelling can compromise the scaffold mechanical properties and likely exert pressure to surrounding tissues, which can hinder vascularisation and provoke tissue necrosis [13]. Despite the higher swelling of DNCs compared to the SNC, which can be attributed to the presence of a second highly hydrophilic network, DNC compressive properties were maintained (Figure 2) due to its denser network. Swelling of DNC-Zn occurred at a slower rate with greater EWC likely due to the smaller ionic size and non-selective binding ability of zinc to alginate dimers that yield a more outstretched and flexible, yet denser network. In contrast, strontium’s larger ionic size and inability to bind to alginate M groups led to sparser and more rigid network which probably caused water molecules to escape, thus the initial water uptake of DNC-Sr was slightly slower than DNC-Ca.

Single glass transition temperature exhibited by DNCs (Table 4) were similar to PVA-alginate copolymer blends [31]; however, the DNC’s T_g_ values between 132–135 °C were higher than the blends, which have been reported to range between 63–64 °C [31]. This suggests that the DN system also increases the complexity of the network entanglement that likely restricts their molecular mobility from glassy to rubbery state. Furthermore, a comparison with a DNC formulated with Bioglass^®^ 45S5 in a similar system [17] exhibited two separate T_g_ slopes associated with PVA (74–86 °C) and alginate (167–218 °C) in contrast to the PVA-CMP DNCs. This can be attributed to the strong interaction of CMP particles with the PVA and alginate networks, possibly via its ubiquitous hydrogen bonds. The T_g_ of the guluronate-dominant alginate chelated by Ca^2+^ was previously observed to be higher than those of Alg-Zn and Sr [16]; however, in this study, DNCs did not show any statistically significant difference in T_g_ among different ions, likely due to the low alginate content compared to PVA. The melting temperature (T_m_) of the composites remained unaffected by the second network (Table 4), as only crystalline materials exhibit melting phase, and alginate is a fully amorphous polymer.

The seamless interface between CMP filler particles and the polymer phase seen in the scanning electron micrographs (Figure 4A) was responsible for efficient dissipation of energy, which improved the mechanical properties of the composites. In the µCT analysis, a denser structure of DNC’s (Table 5) and lower total and open porosity (Table 4) compared to the SNC were attributed to the presence of the alginate network. Since Sr^2+^ exhibits selective binding to GG alginate dimers, the crosslinking nodes become limited, and with the larger ionic size, the uncrosslinked alginate chains likely undergo collapse and entanglement, leading to a denser network. In contrast, zinc ions are smaller in ionic size and able to bind to all alginate dimers, thus decreasing the overall porosity of DNC-Zn. Ca^2+^ on the other hand, can bind to all alginate dimers, except MM blocks, and its ionic size between Zn^2+^ and Sr^2+^ collapse and entanglement of the fewer unchelated alginate chains, thus exhibits a higher porosity than DNC-Sr or DNC-Zn. The near perfect isotropy of the scaffolds enables them to withstand loads evenly.

The threshold of Zn^2+^ cytotoxicity (>100 µM) [32] is known to be much lower than that of Ca^2+^ (>10 mM) [33] and Sr^2+^ (>6.64 mM) [34], although it also depends on the cell types and experimental conditions. However, static in vitro cell culture conditions may exaggerate the toxic effect in comparison to in vivo environment that regularly eliminates any toxic substance and maintains homeostasis. Hence, the reduction in viability observed in 100% DNC-Zn may not be observed in vivo, which can only be confirmed in future in vivo studies.

It is known that a higher surface charge enables higher protein–material affinity [35]. CMP adsorbed higher amounts of protein compared to the composites (Figure 5B), likely due to the openly exposed Ca^2+^ and PO_4_^3−^ in CMP increasing the surface charge, in contrast to the composites whose matrices encapsulate the CMP particles, lowering their surface charge. The higher degradation rate of CMP likely also lowered the pH, thus increasing the ionic strength of the solution and subsequent protein adsorption. The non-selective nature of Zn^2+^ to bind all alginate dimers increases the amount of ions incorporated in the DNC-Zn, which would increase its net surface charge, thus more proteins were adsorbed (Figure 5B). Furthermore, higher swelling of DNC-Zn than DNC-Ca and Sr (Table 3) may also cause higher protein uptake due to larger surface area and simultaneous protein ‘absorption’ with water molecules [36]. Due to higher protein adsorption capacity of the CMP (Figure 5B), the number of initially adhered cells on CMP were higher compared to DNC’s. The proliferation value was high in the early period before reaching a plateau, indicating a phenomenon called ‘density-dependent inhibition of growth’ [37], where the cell proliferative phase ceases as cells reached confluency.

Despite the lower proliferation value (Figure 5C), the total synthesised protein per cell on DNC’s with osteogenic ions increased (Figure 5D), showing intracellular activities beyond proliferative purpose, and towards osteogenic maturation. The drop of the total protein production on day 3 and 7 possibly indicated a ‘growth arrest’ theorised by Lian and Stein, which is a transitory period between the decline of proliferative stage and the start of osteoblastic differentiation in stem cells [38]. The ALP and OCN productions on DNC’s did not follow the widely known concepts where the ALP is normally produced at an early period of osteoblastic differentiation whilst OCN at intermediate to later stage [39]. However, prior to inferring any definitive conclusion, further investigations with larger sample size and/or in vivo studies are required. Furthermore, future studies to explore the antimicrobial properties of DNC’s are recommended, and since properties of biomaterials affect host immune response [40], as well as can potentially induce oxidative stress in the local microenvironment, which may impair the cell growth and subsequent tissue repair [41], it will be of interest to assess the immunogenic response and oxidative stress level induced by these scaffolds.

## 5. Conclusions

A new class of 3D porous hydrogel scaffolds of dual network composites (DNCs) with osteogenic ions: Ca^2+^, Zn^2+^ and Sr^2+^ were successfully designed using non-toxic, simple, low-cost materials and methods. XRD, ^13^C-NMR, FTIR, Raman and DSC results showed that each constituent of the composites was physico-chemically distinct, yet integrated via hydrogen bonds. DNC’s elastic mechanical properties render these DNCs to be easily shaped and inserted into different anatomical bone defects. DNCs can also maintain structural integrity under repeated compressive load, and are thus suitable for use as bone substitutes in critical-sized defects and areas subjected to functional loads, such as the oral and maxillofacial region. They can absorb and retain biological fluids with controlled swelling to support the transport of nutrients and to act as potential concurrent vehicles of biofunctional agents through their hydrogel matrices. Their morphology resembles a trabecular bone with high interconnectivity and isotropy, allowing the cells to thrive and the scaffolds to evenly dissipate energy from multidirectional loads. In vitro biological tests demonstrated that DNC’s were non-cytotoxic, osteoconductive and osteoinductive and are able to promote hBMSC protein adsorption, proliferation, total protein production, upregulate osteogenic gene expressions: RUNX2, BMP2, COL1A1, OPN and/or IBSP, and increase osteogenic protein production: ALP and/or OCN. This study has demonstrated promising results of DNC’s as potentially viable bone substitute materials for oral, maxillofacial and other bone defects, and are thus encouraged to be taken forward to in vivo animal studies.

## Figures and Tables

**Table 1:** Differential scanning calorimetry (DSC) thermal steps and the purpose of each stage.

No.	Action	Target Temperature	Duration/Rate	Purpose
1	Hold	110 °C	10 min	To evaporate the water molecules in the sample without decomposing its constituents (reported temperature of PVA thermal decomposition was 280–290 °C [18])
2	Cool	0 °C	100 °C/min	Rapid cooling
3	Heat	110 °C	10 °C/min	To erase the thermal history of the sample
4	Cool	−10 °C	100 °C/min	Rapid cooling
5	Hold	−10 °C	5 min	To stabilise the baseline, avoiding disrupted curve
6	Heat	300 °C	10 °C/min	To get a clean curve for phase examination
7	Cool	30 °C	100 °C/min	Rapid cooling to finish the test

**Table 2:** Forward and reverse primer sequences for qPCR.

Gene	Forward Primer (5′ 3′)	Reverse Primer (5′ 3′)
GAPDH	ACAGTTGCCATGTAGACC	TTGAGCACAGGGTACTTTA
RUNX2	AAGCTTGATGACTCTAAACC	TCTGTAATCTGACTCTGTCC
BMP2	TCCACCATGAAGAATCTTTG	TAATTCGGTGATGGAAACTG
COL1A1	GCTATGATGAGAAATCAACCG	TCATCTCCATTCTTTCCAGG
OPN	GACCAAGGAAAACTCACTAC	CTGTTTAACTGGTATGGCAC
IBSP	GGAGACTTCAAATGAAGGAG	CAGAAAGTGTGGTATTCTCAG

**Table 3:** Equilibrium water content (EWC) of dual network composites chelated with Ca^2+^ (DNC-Ca), Zn^2+^ (DNC-Zn) and Sr^2+^ (DNC-Sr) compared to the single network composite (SNC) in deionised water at 37 °C. Data are displayed as mean ± SD (*n* = 3). Statistical significance was verified with one-way ANOVA and post hoc Tukey’s analysis (ns: not significant, * *p* < 0.05 and ** *p* ≤ 0.01).

Composite	EWC (%)	Statistical Significance		
		DNC-Ca	DNC-Zn	DNC-Sr
SNC	34.4 ± 1.8	0.0755 (ns)	0.0033 (**)	0.0319 (*)
DNC-Ca	37.3 ± 0.9		0.1606 (ns)	0.9252 (ns)
DNC-Zn	39.6 ± 0.3			0.3572 (ns)
DNC-Sr	37.6 ± 1.2			

**Table 4:** (A) Glass transition (T_g_) and (B) melting temperatures (T_m_) of dual network composites chelated with Ca^2+^ (DNC-Ca), Zn^2+^ (DNC-Zn) and Sr^2+^ (DNC-Sr) compared with the single network composite (SNC). Data are displayed as mean ± SD (*n* = 3). Statistical significance was verified with one-way ANOVA and post hoc Tukey’s analysis (ns: not significant, ** *p* ≤ 0.01 and *** *p* ≤ 0.001).

**A**	**T_g_ (°C)**	**Statistical Significance (*p*-Value)**		
		**DNC-Ca**	**DNC-Zn**	**DNC-Sr**
SNC	124.2 ± 0.7	0.0098 (**)	0.0014 (**)	0.0008 (***)
DNC-Ca	132.0 ± 0.9		0.4200 (ns)	0.2046 (ns)
DNC-Zn	134.9 ± 3.6			0.9375 (ns)
DNC-Sr	135.9 ± 2.2			
**B**	**T_m_ (°C)**	**Statistical Significance (*p*-Value)**		
		**DNC-Ca**	**DNC-Zn**	**DNC-Sr**
SNC	228.8 ± 0.6	0.8569 (ns)	0.1270 (ns)	0.1631 (ns)
DNC-Ca	228.6 ± 0.4		0.3806 (ns)	0.4448 (ns)
DNC-Zn	228.1 ± 0.1			0.9977 (ns)
DNC-Sr	228.1 ± 0.1			

**Table 5:** (A) Object volume/total volume (Obj.V/TV), (B) total porosity, (C) open porosity, (D) open/total porosity and (E) degree of anisotropy of dual network composites chelated with Ca^2+^ (DNC-Ca), Zn^2+^ (DNC-Zn) and Sr^2+^ (DNC-Sr) compared to the single network composites (SNC) in the hydrated state quantified from micro-computed tomography (µCT) results. Data are displayed as mean ± SD (*n* = 3). Statistical significance was verified with one-way ANOVA and post hoc Tukey’s analysis (ns: not significant, * *p* < 0.05 and ** *p* ≤ 0.01).

**A**	**Obj.V/TV (%)**	**Statistical Significance (*p*-Value)**		
		**DNC-Ca**	**DNC-Zn**	**DNC-Sr**
SNC	60.7 ± 7.9	0.1088 (ns)	0.0243 (*)	0.0062 (**)
DNC-Ca	72.5 ± 7.9		0.7152 (ns)	0.2289 (ns)
DNC-Zn	77.2 ± 6.8			0.7336 (ns)
DNC-Sr	81.8 ± 2.0			
**B**	**Total Porosity (%)**	**Statistical Significance (*p*-Value)**		
		**DNC-Ca**	**DNC-Zn**	**DNC-Sr**
SNC	39.3 ± 7.9	0.1088 (ns)	0.0243 (*)	0.0062 (**)
DNC-Ca	27.5 ± 2.5		0.7152 (ns)	0.2289 (ns)
DNC-Zn	22.8 ± 6.8			0.7336 (ns)
DNC-Sr	18.2 ± 2.0			
**C**	**Open Porosity (%)**	**Statistical Significance (*p*-Value)**		
		**DNC-Ca**	**DNC-Zn**	**DNC-Sr**
SNC	36.5 ± 8.7	0.1450 (ns)	0.0276 (*)	0.0075 (**)
DNC-Ca	24.3 ± 3.3		0.6539 (ns)	0.2132 (ns)
DNC-Zn	18.4 ± 7.7			0.7639 (ns)
DNC-Sr	13.4 ± 2.1			
**D**	**Open/Total Porosity**	**Statistical Significance (*p*-Value)**		
		**DNC-Ca**	**DNC-Zn**	**DNC-Sr**
SNC	0.93 ± 0.04	0.9945 (ns)	0.9978 (ns)	0.1162 (ns)
DNC-Ca	0.88 ± 1.31		0.9732 (ns)	0.0825 (ns)
DNC-Zn	0.81 ± 1.14			0.1489 (ns)
DNC-Sr	0.74 ± 1.07			
**E**	**Degree of Anisotropy**	**Statistical Significance (*p*-Value)**		
		**DNC-Ca**	**DNC-Zn**	**DNC-Sr**
SNC	0.10 ± 0.04	0.9875 (ns)	0.9945 (ns)	0.1167 (ns)
DNC-Ca	0.08 ± 0.03		0.9393 (ns)	0.0740 (ns)
DNC-Zn	0.12 ± 0.03			0.1636 (ns)
DNC-Sr	0.26 ± 0.14			
